# Aerosol generation and characterization of multi-walled carbon nanotubes exposed to cells cultured at the air-liquid interface

**DOI:** 10.1186/s12989-016-0131-y

**Published:** 2016-04-23

**Authors:** William W. Polk, Monita Sharma, Christie M. Sayes, Jon A. Hotchkiss, Amy J. Clippinger

**Affiliations:** 1 Integrated Laboratory Systems, Inc, Contractor Supporting the National Toxicology Program Interagency Center for the Evaluation of Alternative Toxicological Methods, Research Triangle Park, NC USA; 2 PETA International Science Consortium Ltd, London, UK; 3 Department of Environmental Science, Baylor University, Waco, TX USA; 4 The Dow Chemical Company, Midland, MI USA; 5 PETA International Science Consortium Ltd, London, UK

**Keywords:** Aerosol engineering, Cell culture model, Exposure, Air-liquid interface, ALI, MWCNTs, Characterization

## Abstract

Aerosol generation and characterization are critical components in the assessment of the inhalation hazards of engineered nanomaterials (NMs). An extensive review was conducted on aerosol generation and exposure apparatus as part of an international expert workshop convened to discuss the design of an in vitro testing strategy to assess pulmonary toxicity following exposure to aerosolized particles. More specifically, this workshop focused on the design of an in vitro method to predict the development of pulmonary fibrosis in humans following exposure to multi-walled carbon nanotubes (MWCNTs). Aerosol generators, for dry or liquid particle suspension aerosolization, and exposure chambers, including both commercially available systems and those developed by independent researchers, were evaluated. Additionally, characterization methods that can be used and the time points at which characterization can be conducted in order to interpret in vitro exposure results were assessed. Summarized below is the information presented and discussed regarding the relevance of various aerosol generation and characterization techniques specific to aerosolized MWCNTs exposed to cells cultured at the air-liquid interface (ALI). The generation of MWCNT aerosols relevant to human exposures and their characterization throughout exposure in an ALI system is critical for extrapolation of in vitro results to toxicological outcomes in humans.

## Background

Multi-walled carbon nanotubes (MWCNTs) are used in a number of consumer products, increasing the potential for inhalation exposure of these materials. Studies suggest that MWCNTs may pose a respiratory hazard to humans (e.g., the development of pulmonary fibrosis), depending on their physico-chemical characteristics [1–7]. Because of the possible link between inhalation exposure of MWCNTs and respiratory toxicity [8], there is interest in better understanding the mechanisms by which MWCNTs can induce inhalation toxicity. Regulatory agencies, such as the U.S. Environmental Protection Agency (EPA) Office of Pollution Prevention and Toxics (OPPT), recommend a 90-day rat inhalation test for MWCNTs if they present or may present an unreasonable risk to human health or the environment (as defined under Section 5 of TSCA) and are projected to be commercially produced.

Due to the substantive time, cost, and animal numbers required to conduct traditional in vivo inhalation toxicity tests there is much interest in in vitro methods to assess the toxicity of these materials. In vitro models have shown promise as human-relevant alternatives to animal testing, and have the capability to rapidly screen the vast number of substances that need to be assessed for toxicity. In vitro tests range from simple acellular or cellular high throughput tests to more complex tests, including three-dimensional (3-D) co-culture systems that better recapitulate the biology, physiology, and exposure dynamics that occur in human airways. An example of a simpler in vitro model for testing the lung effects of NMs could be submerged cultures of a single cell type, and an example of a more complex system could be a co-culture system of relevant cell types exposed to aerosolized NMs at the air-liquid interface (ALI). The two test types are complementary to one another in capability and relevance, offering high throughput (submerged cultures) and the potential for simulating relevant dosimetry with enhanced extrapolatability to the in vivo condition (3-D ALI cultures) when combined in a tiered testing strategy.

Inhalation toxicity was the subject of an international workshop held in Washington, D.C., USA on February 24–25, 2015. The workshop focused on the development of an in vitro test to predict the development of pulmonary fibrosis in cells exposed to aerosolized MWCNTs (Clippinger et al., this journal issue). Based on a literature review and workshop discussions, an in vitro system to assess pulmonary fibrosis following MWCNT exposure should consider aspects such as the mode of exposure (e.g., aerosol exposure of ALI cultures versus liquid media exposure of submerged cultures), relevant receptor (e.g., choice of cell types), concentration, and duration of exposure (e.g., relevance to real exposure scenarios). Characterization at multiple points throughout the MWCNT lifecycle is also important to understand the human relevance of the NM form present in the in vitro system. Presented below are the considerations related to conducting in vitro toxicological studies of aerosolized MWCNTs, including the apparatus and characterizations relevant to the development of an in vitro model to predict pulmonary fibrosis by monitoring pro-fibrotic signals in cells cultured at the ALI following exposure to MWCNTs.

### Nanomaterial targets in the airways

When aerosolized, NMs can exhibit particle characteristics (e.g., size, density, and orientation) that determine their distribution and penetration into different areas of the respiratory system. The respiratory system can be divided into three main regions — nasopharyngeal, tracheobronchial, and alveolar — and is composed of more than 40 types of specialized cells. The alveoli are in a privileged location only accessible by particles with small aerodynamic diameter (usually ranging in size from 0.01–1 μm), are the primary site of gas exchange, and exhibit unique physiology not recapitulated in other parts of the airway [9]. The nasopharyngeal and tracheobronchial regions that constitute the conducting airways are covered by an extracellular fluid layer containing secreted high molecular weight glycoproteins (mucins) and watery secretions, which facilitate the removal of deposited particles (>10 μm) via the mucociliary escalator, active immunocyte surveillance, recruitment, and phagocytosis. Unlike the conducting airways, the alveoli only have a very thin (10–20 nm) epiphase consisting principally of surfactant, serum, and secreted proteins and lack mucociliary clearance allowing for longer retention of particles that reach the alveoli [10–12]. Additionally, the conducting airways limit systemic translocation of particles due to their thick epithelial barriers with deep, torsional channels, ability to slough damaged surface epithelial cells without loss of barrier integrity, and separation of blood from the airway by smooth muscle and connective tissue. Comparatively, alveoli are a very thin tissue barrier (~21 μm), exhibiting the shortest distance in the body between blood and the atmosphere [9, 13–17].

While significant advancements have been made to understand the role of alveoli in disease processes and outcome, much remains unknown because of the inherent limitations of the current test models. Therefore, there has been significant investment in the development of in vitro lung models that include relevant cell types cultured and exposed under physiologically relevant conditions. For instance, it has been suggested that the exposure of alveolar cells cultured at the ALI, in conjunction with other relevant cell types (such as macrophages and fibroblasts), is an ideal model to evaluate the inhalation toxicity of NMs [18]. Additionally, in order for such a model to accurately reflect human-relevant in vivo exposure, the mode of administration of NMs should be carefully considered. Thus, it is critical to evaluate the suitability of available cell culture systems for studying NMs as well as the techniques for aerosolizing and exposing NMs to cells cultured at the ALI.

### Exposure methods

Cell cultures can be exposed to aerosolized materials by direct and indirect methods. Indirect exposure methods for aerosol studies typically involve collection of aerosols on sampling substrates (e.g., filter) or collection apparatus (e.g., wetted wall cyclones, wetted rotating vane impactors, or liquid impingers), followed by recovery of the collected aerosols and suspension of solid aerosols in culture medium before exposure of cells in a submerged culture [19, 20]. This indirect exposure technique has been used to investigate biological effects of a wide range of test materials from NMs to near-road ambient particles [21–23]. Although such techniques offer advantages for the testing of aerosols collected on-site, the intermediate steps, such as extraction of collected aerosols from the sample collection matrix and solubilization in exposure medium, could potentially alter the aerosol form and lead to false assessments.

Direct exposure methods are used for gas-phase exposure of test aerosols using several exposure techniques. Direct exposure methods enable administration of aerosolized materials directly to cultured cells under submerged conditions, intermittent exposure by a rocker platform, or continuous exposure at the ALI, among others [20, 24]. Direct continuous exposure at the ALI is more representative of human-relevant exposures than intermittent exposure and submerged systems, and it can be used to assess the potential hazards associated with aerosolized materials in a scalable, flexible, dosimetrically-relevant format that addresses the challenges put forth for modernizing toxicology testing [25].

In an ALI system, target cells are cultured on permeable and porous cell culture inserts (e.g., Transwell® inserts) and exposed to test atmospheres continuously on the apical surface, simulating the contact of pulmonary cells with inhaled air, while being nourished with medium from the basolateral surface. The ALI system provides a larger interface between airborne components and target cells as compared to the intermittent exposure or submerged exposure conditions. Additionally, ALI systems can limit the transformation of NM properties due to their interaction with the medium because the depth of the liquid on the surface of the ALI cell layer is significantly lower in comparison to submerged cultures. This is important because the transformations that NMs undergo in the exposure medium may influence the accuracy and reproducibility of the exposure. For example, one study showed aerosolized particles exposed at the ALI of a 3-D lung cell model to undergo far less agglomeration and thus less sedimentation compared to submerged cultures [26].

**Fig. 1:**
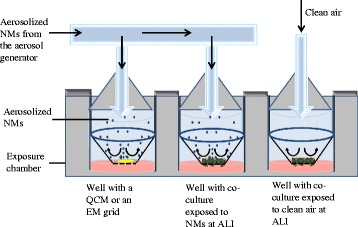
Basic configuration of a system for exposing cells to aerosolized substances at the ALI. Such a system requires an aerosol generator and an exposure chamber. Clean air controls may be incorporated as well as a means to quantify the cellular dose (e.g., using a quartz crystal microbalance (QCM) and/or electron microscopy (EM) grids) to relate the outcomes to human-relevant exposures

Development of ALI systems that recapitulate the human lung is ideal for testing MWCNTs, or other substances that may be inhaled, for their potential to cause fibrosis. While less complex systems, such as submerged cell cultures, are useful in understanding toxicity, ALI exposure of primary human cells cultured in a physiologically relevant 3-D configuration can aid in human risk assessment. Exposure of cells to aerosolized substances at the ALI requires an aerosol generator (to generate the test MWCNT atmosphere) and an exposure chamber (to deliver the aerosolized test MWCNT atmosphere to the cells at ALI). Figure 1 shows the basic configuration for an ALI exposure system. The aerosol generators and exposure chambers that are available to use with MWCNTs are discussed in the following sections.

### Aerosol generation of MWCNTs

There are several methods commonly used for aerosol generation that differ in the basic principle used to generate the aerosols. Oberdörster et al. provides a comprehensive review of the generator and exposure systems that may be adapted to in vitro systems [27]. The physical characteristics of the material to be aerosolized, such as density, viscosity, state of matter, and target aerosol size, determine which generator should be used. MWCNTs can be delivered to cells either as a dry solid (dust) or liquid particle suspension aerosol. For liquid or biological materials, collision nebulizers, jet nebulizers, ultra-sonic atomization, or vibrating membrane generators are often used; whereas, the generation of aerosolized solid materials have typically employed the use of spray-drying, rotating scrapers, venturi-style powder dispersions, or fluidized powder bed methods [28–31]. When MWCNTs are delivered in a suspension (for instance, when using a nebulizer), it is important to understand if the suspension or carrier solvent contributes to the observed biological effect. Use of a dry aerosol generator (e.g., acoustic) allows for the evaluation of the relevance of the deposited form and concentration to the realistic exposure. When using nebulization, MWCNTs can be suspended in simulant lung surfactant; for dry aerosol generation methods, a cell line that secretes lung surfactant can be used to further simulate physiological conditions.

Descriptions of commonly used aerosol generation methods are shown in Table 1 as well as the type, size, concentration, and duration of aerosol generated using each. Each of the techniques has strengths and weaknesses depending on the nature of the material to be aerosolized, the particle size and concentration needed, and the duration of exposure. For instance, to assess the risk to humans, the ideal aerosol generator would be capable of generating MWCNTs in the human respirable size range (that is, particles that enter the lower respiratory tract and have a mass median aerodynamic diameter (MMAD) of between 1 and 3 microns) [32, 33]. Therefore, an aerosol generator should be carefully selected based on its suitability with the chosen MWCNT type and the requirements of the study design.

**Table 1:** Aerosol generators

Type	Principle	Material type	Size range	Maintainable duration	Concentration range	Ref
Nebulizer	Droplets are formed with an atomizer or in a fountain formed by ultrasonic sound. Dried droplet residue forms the particles.	Liquids containing dissolved or suspended solids	0.43–16.2 μm	Optimal for 6-h durations	0.01–5 mg/m^3^	[34]
Electro spray Generator	High voltage is applied to a metal capillary end containing flowing liquid. Expelled charged droplets fragment when electrostatic forces exceed surface tension. Dried droplet residue forms the particles.	Liquids containing dissolved or suspended solids	2- 100 nm	Optimal for 4 h	5 ng/cm^2^/min for in vitro and 2 mg/m^3^ for in vivo	[35]
Fluidized bed	Small beads are fluidized by air, and the turbulent motion and bead interaction disperses powder added to the bed.	Solid	20–500 μm	Greater than 3 h	0.5–40 mg/m^3^	[31], [36]
Acoustic	Includes an acoustic energy source and a diaphragm(s) that produce a pressure gradient.	Solid	Same as the original particle size	Greater than 30 h	15 mg/m^3^ or more	[37]
Brush feed	Composed of a screw feeder, rotating brush, and a cyclone designed to remove larger particles to aerosolize carbon fibers.	Solid	Same as the original particle size	In 6 h increments	5 mg/m^3^	[38]
Dust feeder	The surface of a cake of compacted powder is scraped at a controlled rate, by mechanical scraping and blown by compressed air.	Solid	Same as the original particle size	>20 h	Concentration of the output aerosol can be controlled by adjusting the air-flow rate	[27, 39], [40]

The table provides a brief description of aerosol generators that are applicable to MWCNTs, including the principle of aerosol generation, the type of material that can be aerosolized, the size and concentration of the aerosolized particle, and the duration of exposure to the aerosolized particles

### Exposure chambers for MWCNT aerosolization

Chambers that can be used to expose cells cultured at the ALI to aerosolized materials have been described in the literature and include both commercially available systems and those developed by independent researchers [40, 41]. These chambers differ from each other in factors, such as cost, ease of availability and use, level of throughput, exposure time duration, and the number of examples of use in the published literature. Based on these factors, chambers from three organizations — VITROCELL® Systems, Cultex® Laboratories GmbH, and the University of Bern — were selected as most relevant for testing MWCNTs (Table 2). The chambers from VITROCELL® and Cultex® are commercially available, while the chamber from the University of Bern is produced in bulk (five at a time) by an independent researcher and is available on demand.

**Table 2:** Characteristics of air-liquid interface exposure chambers for in vitro cell cultures

	VITROCELL^®^ 6/6, 12/12, OR 24/24	VITROCELL^®^ Cloud 6, 12, OR 24	Cultex^®^ RFS OR RFS COMPACT	University of Bern NACIVT
What level of throughput can be achieved?	VITROCELL^®^ 6/6	VITROCELL^®^ Cloud 6	Cultex^®^ RFS	NACIVT
	• 6-well inserts (can use adaptors to adapt to 12 or 24-well-sized)	• 6-well inserts	• 6, 12, or 24-well inserts	• 6-well inserts
	• compartments for exposing 6 cell culture inserts	• compartments for exposing 6 cell culture inserts: 6 inserts at one exposure; no clean air control	• compartments for exposing 3 cell culture inserts - all exposed to the same substance	• compartments for exposing 24 cell culture inserts – allexposed to the same substance.
	• allows for separate clean air exposure in one module:	• 1 well can be used for the (optional) microbalance.	• would have to purchase 2 modules for clean air control	• would have to purchase 2 modules for clean air control
	o 3 replicates clean air control			
	o 3 replicates of one dose (or 2 replicates plus optional 1 well for microbalance)			
	VITROCELL^®^ 12/12	VITROCELL^®^ Cloud 12	Cultex^®^ RFS Compact	
	• 12-well inserts (can use adaptors to adapt to 24-well-sized)	• 12-well inserts	• 12 or 24-well inserts	
	• compartments for exposing 12 cell culture inserts	• compartments for exposing 12 cell culture inserts	• compartments for exposing 6 cell culture inserts	
	• allows for separate clean air exposure in one module:	• allows for separate clean air exposure in one module:	• allows for separate clean air exposure in one module	
	o 3 replicates clean air control	o 3 replicates for clean air control		
	o 3 dose dilutions with 3 replicates per dilution (or 2 replicates plus optional 1 well for microbalance)	o 9 inserts at one exposure (1 well can be used for the optional microbalance)		
	VITROCELL^®^ 24/24	VITROCELL^®^ Cloud 24		
	• 24-well inserts	• 24-well inserts		
	• compartments for exposing 24 cell culture inserts	• compartments for exposing 24 cell culture inserts; 24 inserts at one exposure (1 well can be used for the optional microbalance)		
	• allows for separate clean air exposure in one module:	• would have to purchase 2 modules for clean air control		
	o 4 replicates clean air control			
	o 5 dose dilutions with 4 replicates per dilution (or 3 replicates plus optional 1 well for microbalance)			
Commercially available?	Yes	Yes	Yes	No
Deposited-dose determination	• The microbalance is capable of measuring the deposition in the module at a resolution of 10 ng/cm^2^.	1 well can be used for the microbalance (optional).	Gravimetric methods, using the precision balance.	One of the wells is designed to carry a transmission electron microscope (TEM) grid to monitor exposure. Also has an electrometer.
	• For the 6/6 and 12/12, a built-in microbalance option is available. A microbalance sensor would occupy one compartment; therefore, the module should have at least 4 compartments in order to allow 3 replicates for exposure.			
	• For the 24/24, the purchase of a separate, standalone 12/1 CF module to use a microbalance would be needed.			
Exposure Method	The VITROCELL^®^ 6/6 is compatible with electrostatic deposition.	Not compatible with electrostatic deposition, but due to high deposition efficiency of NMs in the Cloud, electrostatic deposition is not needed (Cloud is equipped with a nebulizer that generates liquid aerosols).	Compatible with electrostatic deposition (both RFS and RFS compact).	Compatible with electrostatic deposition.
	The 12/12 and 24/24 are not, but in those cases, the VITROCELL^®^ 6/6 modules can be used with adaptors for 12 or 24-well inserts.			
Exposure time duration	Maximum 6 h possible	Approximately 3–4 min	Up to 1 h reported	Up to 2 h reported
Ability to utilize multiple aerosol generating techniques?	May be used with a wide range of aerosol generators.	Compatible only with the aerosol generator, which is part of the delivery (vibrating mesh type for liquid aerosols).	May be used with a wide range of aerosol generators; One option is the Cultex^®^ dust generator, which is a Mitchell or screw mill type.	May be used with a wide range of aerosol generators; Can be placed close to the workplace or aside a busy street.

VITROCELL® Systems offer multiple chambers, including the ‘original’ (VITROCELL® 6, 12, and 24) and the Cloud (VITROCELL® Cloud 6, 12, and 24) set-ups. Cultex® Laboratories offer two chambers: the Radial Flow System (RFS) and the RFS Compact. The University of Bern manufactures an exposure module called the Nano Aerosol Chamber for In-Vitro Toxicity (NACIVT). Importantly, organizations frequently update existing modules or add new modules to their product line, and the information below is based on the status at the time of this publication. The chambers reviewed have several common features, including their compatibility with commercially available cell culture inserts and multiple aerosol generators, a mechanism to regulate temperature during exposure, and the ability to expose cells to aerosolized MWCNTs. However, there are differences that make each system unique, and these differences are detailed in the following sections.

#### Basic chamber configuration

Testing for the purpose of hazard assessment or identification requires specific attention to the number of replicates, appropriate controls, and quality assessment of the experiment. As the available aerosol exposure chambers exhibit limited end-user flexibility to modify the system configuration, the general need of the experiment must be assessed before deciding which system to use. For example, consideration should be given to whether the exposure chamber is: (1) capable of consistently delivering and depositing MWCNT aerosols at multiple dilutions; (2) compatible with different types of aerosol generators; (3) capable of determining the deposited dose; (4) readily cleaned; and (5) regularly available and consistently reproduced to facilitate interlaboratory transferability. Furthermore, the size of the cell culture inserts, the number of wells, and the number of different exposure concentrations that could simultaneously be tested per exposure system should be considered. To understand mechanisms of complex pathological outcomes, it is critical to focus on specific inter- and intra-cellular biomarkers (e.g., monitor multiple pro-fibrotic markers to understand the development of fibrosis), which will require a larger number of cells per exposure than are normally cultured in high throughput wells. Similarly, establishing a dose-response requires testing multiple concentrations of the test substance in one experiment, and therefore, it would be ideal to have a chamber with separate modules to accommodate different exposure concentrations (doses).

The ability to run a clean air exposure simultaneously with the MWCNT is ideal to associate the changes in biological responses to the test material. Some of the chambers (including VITROCELL® 6/6, 12/12, 24/24, Cloud 12, and Cultex® RFS Compact) provide the option of running clean air control experiments at the same time as the test material. For the chambers not capable of running simultaneous clean air control experiments (including VITROCELL® Cloud 6 and 24 Cultex® RFS, and NACIVT), either separate modules need to be purchased (one for the clean air control and one for the test substance), or if only one module is available, the clean air control experiment and test material experiment have to be run separately. This will double the time needed to conduct the study and will require that the module and auxiliary equipment are thoroughly cleaned between experiments to remove all traces of MWCNTs. Overall, the focus and goal of the study should be considered when choosing the exposure chamber. Although this review focuses on the three chambers (VITROCELL®, Cultex®, and NACIVT), the parameters described would also be relevant for the evaluation of other chambers.

#### Modularity

The exposure systems from the three manufacturers differ in their flexibility of peripherals and connections, with VITROCELL® Systems (with the exception of the VITROCELL® Cloud) being more modular in comparison to the Cultex® and NACIVT systems. Although modularity can provide the ability to fine tune the system and to expose different materials or different exposure concentrations (doses) at the same time, these modifications should only be made by someone with appropriate expertise and training since small variations in parameters (such as length of peripheral tubing) can impact the particle size and concentration of the MWCNT exposure atmosphere. The Cultex®, NACIVT, and the VITROCELL® Cloud systems, on the other hand, are built as one unit.

#### NM deposition

While all of the systems described above (except the VITROCELL® Cloud systems) allow for the use of different types of aerosol generators, there are considerable differences between the systems with regards to the duration for which the cells can be exposed to the aerosolized NMs (see Table 2). Exposure duration can significantly affect the amount of MWCNTs generated and deposited onto the cell layer and should therefore be carefully chosen. Large or dense particles in a moving airstream can be readily deposited through settling or impaction, as inertial and gravimetric forces are great enough to overcome thermodynamic and flow forces that would keep the particles suspended. Conversely, low-density particles, like MWCNTs, and those smaller than 100 nm deposit primarily via diffusion [42].

The issue of low deposition can be overcome by electrostatic deposition enhancement, which is a technique that uses charge to increase the efficiency of deposition by several fold (5–10 fold). For some materials, charge must first be added to the particles; however, this step is not necessary for aerosolized MWCNTs, which are extremely charged. The VITROCELL®, Cultex®, and NACIVT are adaptable to electrostatic deposition but dose enhancement is not available for VITROCELL® Cloud because it uses liquid aerosolization (i.e., nebulization) of MWCNTs, which yields high deposition efficiency. Although the physiological relevance of using electrostatic deposition is debatable, specified and reproducible concentrations of MWCNTs can be administered to cells via electrostatic deposition of aerosolized materials or nebulization of MWCNTs dispersed in a relevant medium (e.g., simulant lung surfactant).

#### Monitoring the deposited dose

The exposure chambers described above come with the option of a quartz crystal microbalance (QCM) that allows for a relatively quick and easy way to gauge deposition at a resolution of 10 ng/cm^2^. NACIVT also has the option of an internal aerosol electrometer to measure particle concentration in real time, so that the particle dose administered to the cells can be estimated. Alternative analytical methods to determine the deposited dose of MWCNTs for ALI cell cultures that can be used with all the available chamber systems involve techniques, such as laser spectrometry and electron microscopy (scanning electron microscopy (SEM) and transmission electron microscopy (TEM)) [43, 44].

### Characterization of MWCNTs for assessing pulmonary toxicity using ALI systems

In addition to choosing the appropriate aerosol generation and exposure apparatus, it is critical to characterize MWCNTs to associate the observed endpoint to the causative NM property. Aerosol generation and exposure systems can be equipped with auxiliary equipment to characterize MWCNTs at various time points. There is consensus among researchers that characterization of the administered dose (used here to describe the exposure concentration times the volume of test atmosphere that flows through the ALI chamber) alone is not sufficient as NM properties may change following deposition and cellular uptake. It is therefore important to characterize and understand the MWCNT form throughout its lifecycle.

**Fig. 2:**
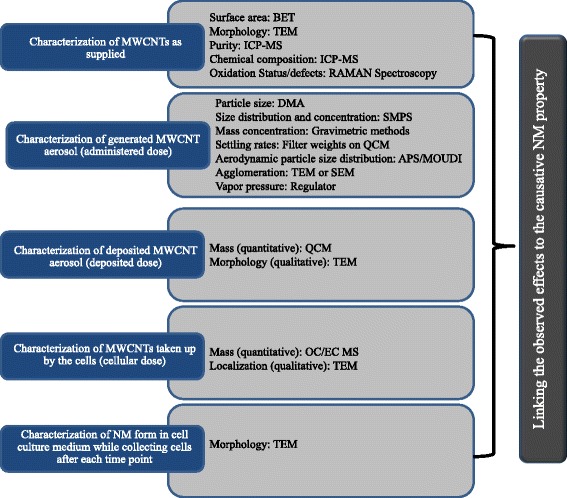
The schematic shows the stages that are most critical for NM characterization and the techniques relevant to MWCNTs. Stage 1 involves characterization of the NM in its pristine form; stage 2 involves characterization of the administered dose; stage 3 is characterization of the deposited dose; stage 4 is characterization of the cellular dose; and stage 5 involves post-exposure evaluation of NM transformations during the course of the assay. Each stage looks at particular NM properties and requires specific techniques. The techniques mentioned in the schematic are Brunauer, Emmett and Teller (BET), scanning electron microscopy (SEM), transmission electron microscopy (TEM), inductively coupled plasma-mass spectroscopy (ICP-MS), differential mobility analyzer (DMA), scanning mobility particle sizer spectrometers (SMPS), aerodynamic particle sizer (APS), micro-orifice uniform deposit impactor (MOUDI), and organic carbon/ elemental carbon (OC/EC) mass spectroscopy

Ideally, physico-chemical properties of MWCNTs would be characterized in five separate stages (Fig. 2). The first stage occurs prior to aerosolization (pristine form/as supplied), the second is the MWCNT atmosphere present within the exposure chamber (administered dose), the third stage is the MWCNTs that settle on the cell surface (deposited dose), and the fourth stage is the MWCNTs taken up by the cells (cellular dose). In addition to the aforementioned stages, the fifth stage involves post-exposure evaluation of MWCNT transformations during the course of the assay, such as after each time point. The MWCNT characteristics that are relevant to some of the aforementioned stages include, but are not limited to: 1) agglomerate structure and (de) agglomeration potential; 2) impurity profile/content; 3) effective density (specific gravity) of the deposited form of the NM; 4) bivariate length and diameter distribution (BVD); 5) surface charge; 6) surface area; 7) rigidity; 8) dustiness; and 9) cellular uptake. While evaluating the MWCNTs at every stage is ideal, it is very resource intensive. Therefore, a list of characteristics should be prioritized to identify the properties of the MWCNTs that are critical to understand the context-specific fate of NMs and their subsequent biological effects. These properties and relevant characterization techniques have been depicted in Fig. 2 and described in the following sections.

#### Stage1: characterization of MWCNTs as supplied

MWCNTs have diverse physico-chemical properties that are dependent on the procedures used to synthesize them and are relevant to their prospective use. Depending on the synthesis process, MWCNTs can be produced in varying sizes (length and diameter), shapes (straight, rigid, bent, curled, and flexible), and with varying surface chemistry (oxidation status, functionalization, and trace metal content). The diverse physico-chemical properties dictate the lifecycle transformations (e.g., agglomeration, oxidation, and degradation) that MWCNTs will undergo, which eventually translate to different biological and ecological impacts of these MWCNTs.

A number of techniques are available to characterize the pristine form of MWCNTs and have been described in the reports published by the European Commission Joint Research Center (JRC) and the United States National Institute for Occupational Safety and Health (NIOSH) [8, 45]. Table 3 indicates the properties characterized for the pristine form of MWCNTs from various manufacturers and the techniques used, as described in the published literature. Physical dimensions, including length and diameter to estimate aspect ratio, are usually determined using electron microscopy based techniques (SEM and TEM). Specific surface area can be determined using the Brunauer, Emmett and Teller (BET) method; x-ray diffraction can be used to detect the crystal structure; EDS coupled with SEM or TEM can be used to detect the composition of MWCNTs and presence of catalysts; and RAMAN spectroscopy can be used to analyze oxidation status and defects.

**Table 3:** Examples of MWCNTs with their characterization information taken from the published literature

MWCNTs type/manufacturer	Characterization		Ref.
MWNT-7 (lot #05072001 K28)/Mitsui & Co., Inc. (USA)		MWCNT	[5, 46–50]
	L (SEM, μm)	3.86	
	D (SEM, nm)	49 ± 13.4	
	Trace metal contamination [sodium (0.41 %) and iron (0.32 %)] (Also reported 1.32 % for total and 1.06 % for iron content)	0.78	
	SSA (BET, m^2^/g)	26	
	Zeta potential (mV)	-11	
	Number of walls	20–50	
Baytubes/Bayer Material Science, (Germany; no longer commercially available)		MWCNT	[1, 50]
	Cobalt content (%, wt/wt) (ICP-OES)	0.46	
	Content of cobalt (Co) (%, wt/wt) (AAS)	0.53	
	Elemental analysis (% carbon-oxygen)	98.6–1.4	
	SSA (BET, m^2^/g)	253	
	Bulk density (g/cm^3^)	0.16	
NM400 and NM402/JRC		NM400	[45, 51, 52]
	L (SEM, μm)	0.7–3	
	D (SEM, nm)	5–35	
	Impurities total, % Al, % Co	5.38 total, 0.24, 0.58	
	(Also reported 13 % for total impurities)		
	Defects	1.2	
	SSA (BET, m^2^/g)	245.8	
NM401/JRC		CNT_Large_	[45, 52]
	L (SEM, TEM, μm)	4.05 ± 2.4	
	D (SEM, TEM, nm)	67 ± 26.2	
	Impurity (%)	3	
	SSA (BET, m^2^/g)	14.6	
NM402/JRC		NM402	[45, 51]
	L (SEM, μm)	0.7–4	
	D (SEM, nm)	6–20	
	Impurities total, % Al, % Co	3.16,	
	(Also reported 13 % for total impurities)	3.00 x 10^-4^, 2.39	
	Defects	1.12	
MWCNTs/Helix Materials Solutions, Inc. (Richardson, TX)		MWCNT	[53–55]
	L (TEM, μm)	0.3–50	
	D (TEM, nm)	10–30	
	Purity (TGA, %)	>94	
	Metal content % Ni, %La (ICP-AES)	0.34 and 0.03 by weight	
	Metal %Ni (EDX)	0.12	
	SSA (BET, m^2^/g)	109	
	Zeta potential (mV)	-13 ± 2	

Characterization parameters such as length (*L*), diameter (*D*), specific surface area (*SSA*), density, trace metal content, and zeta potential are given for different MWCNTs in addition to the techniques (where available) used for measuring each of the parameters. The techniques used include Brunauer, Emmett and Teller (*BET*), scanning electron microscopy (*SEM*), transmission electron microscopy (*TEM*), inductively coupled plasma atomic emission spectroscopy (*ICP-AES*), thermogravimetric analysis (*TGA*), atomic absorption spectroscopy (*AAS*), and energy-dispersive X-ray spectroscopy (*EDX*)

#### Stage 2: administered dose

The administered dose is the MWCNT aerosol atmosphere produced within the exposure chamber at a specific concentration to be tested in the in vitro system. It is analogous to the inhaled dose in an in vivo inhalation exposure study in that it reflects the concentration of the test material and represents the maximal potential dose if all of the test material were deposited. It is important to characterize the administered dose to determine the the physico-chemical composition and form of the test material that is ultimately deposited on cells at the ALI. Chemical properties of the aerosol (e.g., composition, redox potential, and impurities/defects) and the physical features (e.g., particle and mass concentration, settling rates, aerodynamic particle size distribution, and the agglomeration of particles) should be characterized.

Physical features of aerosolized MWCNTs may be measured using a scanning mobility particle spectrometer (SMPS) which includes a differential mobility analyzer (DMA) to determine particle size distribution and a condensation particle counter (CPC) to measure the particle count concentration. An aerodynamic particle sizer (APS) may be used alone or in conjunction with an SMPS to determine aerodynamic properties of the test material. Additionally, a micro-orifice uniform deposit impactor (MOUDI) is used for precise, high accuracy aerosol sampling and for collecting size-fractionated particle samples for gravimetric and/or chemical analysis. The techniques should be chosen based on the NM type; for instance, SMPS might not be appropriate to characterize charged MWCNTs [56]. In addition to aerosol characterization, determination of parameters, such as pressure, temperature, air flow, and relative humidity, inside the exposure-chamber is critical since these parameters can affect the aerosol form that reaches the apical surface of the cells at the ALI.

#### Stage 3: deposited dose

The deposited dose is the total amount of MWCNTs that deposit on the apical cell layer and is different from the administered dose. Determination of the dose-response relationship requires an accurate measurement of the deposited particle mass onto the cell culture. The most common way to determine the deposited dose is to use the sensor of a QCM [57–59]. The QCM is placed in one empty well of the exposure chamber and exposed to the aerosol. The deposited mass per unit area is monitored as a function of exposure time that is used in calculating the particle concentration. In addition to determining the mass of the deposited MWCNTs, it may be critical to express dose based on the number of particles deposited, as fibers, such as asbestos, are measured in terms of the number of particles per unit volume. The number of fibers deposited can be determined using electron microscopy based methods, where a TEM or SEM grid is placed in the well, and the particles can be counted using representative images of the deposited test material to estimate total particle count. In addition to particle mass and number, surface area is another metric that is considered at the Organisation for Economic Co-operation and Development (OECD) level [60].

#### Stage 4: cellular dose

The fourth stage of characterization is determining the form and concentration of internalized cellular dose of the MWCNT. The published literature shows that NM cytotoxicity, including MWCNTs, is proportionally related to the dose (or concentration) in terms of mass or particle number, internalized within individual cells, and averaged among the entire cell population. As internalized dose increases, cytotoxicity also increases [61, 62]. Two instruments can be used to determine cellular uptake: TEM is used as a qualitative assessment, while inductively coupled plasma-mass spectroscopy (ICP-MS) is used as a quantitative measure. In the case of MWCNTs, the internalized dose can also be determined directly by elemental carbon analysis or indirectly by measuring the amount of trace metal impurities associated with the MWCNTs using ICP-MS based techniques [63]. Ashing methods have also been developed for measuring the trace metal content, which can then be used to calculate the total dose [64, 65]. In addition to providing some insight into the total dose delivered, determination of trace metal content is an important analytic output since low concentrations of metals may impact the observed toxicological outcome or confound other results [66].

#### Stage 5: NM transformations

The fifth stage of characterization is measured post-exposure throughout the course of the assay. It is well known that the physico-chemical properties of NMs change when suspended in aqueous media. Particles can undergo changes, such as agglomeration (both homoagglomeration (agglomeration with NMs) and heteroagglomeration (agglomeration with other matrix-specific colloids)), dissolution, and oxidation, as well as changes in surface properties (biodegradation and corona formation). Therefore, collecting aliquots of MWCNTs at different time points in the study is key in evaluating particle transformations [67]. For example, samples should be analyzed using TEM (to record changes in size and/or agglomeration) and ICP-MS (to determine loss of ionic function).

Thorough characterization can help to define parameters specific to MWCNTs for *in silico* modeling. For instance, the Multi-Path Particle Dosimetry (MPPD) model (available from Applied Research Associates, Inc.) and the In vitro Sedimentation, Diffusion, and Dosimetry (ISDD) model have been used to estimate deposited dose based on particle size for interspecies extrapolation of particle exposures in various species and to understand the kinetics of MWCNTs in the in vitro systems [42, 68, 69]. Such models can be used to make predictions regarding MWCNT fate and transport in complex biological systems that can help in designing in vitro systems that are more predictive of in vivo conditions.

## Conclusions

Simulating human-relevant exposure is critical for assessing the toxicological potential of MWCNTs in in vitro systems. When evaluating pulmonary effects (e.g., the development of fibrosis) related to MWCNT exposure for human risk assessment, exposure of relevant cell types (representing the respiratory tract) to aerosolized MWCNTs at the ALI holds greater physiological relevance than traditional submerged cultures. Assessment of inhalation toxicity using cells exposed at the ALI requires an aerosol generator and an exposure chamber. For MWCNTs, all exposure apparatus discussed in this review met the critical needs to establish a robust method, but none address all aspects of an ideal in vitro exposure platform. For example, options not currently included in any model include high throughput capabilities (>10,000 samples per day), available real-time sampling of the basolateral compartment, and generation of fluid shear stress in the basolateral compartment.

The set-up and use of aerosol generation, exposure, and characterization systems requires knowledge about the engineering aspects of aerosol generation and characterization, in addition to the biology of morphologically distinct respiratory tract cells cultured at the ALI. Because of the interdisciplinary nature of the topic, this manuscript fills a critical need of putting forth the parameters that are critical to consider while assessing the inhalation toxicity of MWCNTs using in vitro methods. These parameters include choice of aerosol generation and exposure equipment to study the relevant route and duration of exposure, context-specific characterization of MWCNTs and their transformations at various lifecycle stages, and evaluation of in vitro data in the light of existing information. Although this manuscript focuses on MWCNTs, the technical considerations described here can be applied to the evaluation of inhalation toxicity of other NMs and substances. Development of research strategies based on the parameters evaluated in this manuscript can help in generating comprehensive information on biological endpoints relevant to inhalation exposure to NMs or other larger respirable aerosol particulates, which could be used in the hazard ranking of substances in the risk assessment process.

## Abbreviations

AAS: atomic absorption spectroscopy
Al: aluminum
ALI: air-liquid interface
APS: aerodynamic particle sizer
BET: brunauer, emmett and teller
BVD: bivariate data
Co: cobalt
DLS: dynamic light scattering
DMA: differential mobility analyzer
EDX: energy-dispersive X-ray
EM: electron microscopy
U. S. EPA: United States environmental protection agency
GI: gastrointestinal
ICP-AES: inductively coupled plasma atomic emission spectroscopy
ICP-MS: inductively coupled plasma mass spectroscopy
ICP-OES: inductively coupled plasma optical emission spectroscopy
ILS: integrated laboratory systems, Inc
ISDD: in vitro sedimentation, diffusion, and dosimetry
MMAD: mass median aerodynamic diameter
MPPD: multi-path particle dosimetry
MOUDI: micro-orifice uniform deposition impactor
MWCNT: multi-walled carbon nanotube
NACIVT: nano aerosol chamber for in-vitro toxicity
NICEATM: NTP interagency center for the evaluation of alternative toxicological methods
NIEHS: national institute of environmental health science
NM: nanomaterial
NTP: national toxicology program
OC/EC: organic carbon/elemental carbon
OPPT: office of pollution prevention and toxics
QCM: quartz crystal microbalance
RFS: radial flow system
SEM: scanning electron microscopy
SMPS: scanning mobility particle sizer spectrometer
SSA: specific surface area
TEM: transmission electron microscopy
TGA: thermogravimetric analysis
TSCA: toxic substances control act
